# APOBEC3 enzymes mediate efficacy of cisplatin and are epistatic with base excision repair and mismatch repair in platinum response

**DOI:** 10.1093/narcan/zcaa033

**Published:** 2020-11-06

**Authors:** Kayla L Conner, Asra N Shaik, Katie A Marshall, Ashley M Floyd, Elmira Ekinci, Jacob Lindquist, Akshada Sawant, Wen Lei, Madison B Adolph, Linda Chelico, Sachini U Siriwardena, Ashok Bhagwat, Seongho Kim, Michele L Cote, Steve M Patrick

**Affiliations:** Department of Oncology, Wayne State University School of Medicine and Barbara Ann Karmanos Institute, Detroit, MI 48201, USA; Department of Oncology, Wayne State University School of Medicine and Barbara Ann Karmanos Institute, Detroit, MI 48201, USA; Department of Biology, University of Michigan, Ann Arbor, MI 48109, USA; Department of Oncology, Wayne State University School of Medicine and Barbara Ann Karmanos Institute, Detroit, MI 48201, USA; Department of Oncology, Wayne State University School of Medicine and Barbara Ann Karmanos Institute, Detroit, MI 48201, USA; Department of Oncology, Wayne State University School of Medicine and Barbara Ann Karmanos Institute, Detroit, MI 48201, USA; Rutgers Cancer Institute of New Jersey, 195 Little Albany Street, New Brunswick, NJ 08903, USA; Department of Oncology, Wayne State University School of Medicine and Barbara Ann Karmanos Institute, Detroit, MI 48201, USA; Vanderbilt University, Nashville, TN 37232, USA; Department of Biochemistry, Microbiology and Immunology, College of Medicine, University of Saskatchewan, 103 Hospital Drive, Saskatoon, SK S7N 5E5, Canada; Department of Chemistry, Wayne State University, Detroit, MI 48202, USA; Department of Biochemistry, Microbiology and Immunology, Wayne State University School of Medicine, Detroit, MI 48201, USA; Department of Chemistry, Wayne State University, Detroit, MI 48202, USA; Department of Biochemistry, Microbiology and Immunology, Wayne State University School of Medicine, Detroit, MI 48201, USA; Department of Oncology, Wayne State University School of Medicine and Barbara Ann Karmanos Institute, Detroit, MI 48201, USA; Department of Oncology, Wayne State University School of Medicine and Barbara Ann Karmanos Institute, Detroit, MI 48201, USA; Department of Oncology, Wayne State University School of Medicine and Barbara Ann Karmanos Institute, Detroit, MI 48201, USA

## Abstract

Identifying the mechanisms mediating cisplatin response is essential for improving patient response. Previous research has identified base excision repair (BER) and mismatch repair (MMR) activity in sensitizing cells to cisplatin. Cisplatin forms DNA adducts including interstrand cross-links (ICLs) that distort the DNA helix, forcing adjacent cytosines to become extrahelical. These extrahelical cytosines provide a substrate for cytosine deaminases. Herein, we show that APOBEC3 (A3) enzymes are capable of deaminating the extrahelical cytosines to uracils and sensitizing breast cancer cells to cisplatin. Knockdown of A3s results in resistance to cisplatin and induction of A3 expression in cells with low A3 expression increases sensitivity to cisplatin. We show that the actions of A3s are epistatic with BER and MMR. We propose that A3-induced cytosine deamination to uracil at cisplatin ICLs results in repair of uracils by BER, which blocks ICL DNA repair and enhances cisplatin efficacy and improves breast cancer outcomes.

## INTRODUCTION

Cisplatin is a platinum-based chemotherapeutic used in treating multiple types of cancer, including lung cancer and head and neck cancer (1). Cisplatin is a DNA damaging agent that forms mono-adducts, intrastrand adducts and interstrand cross-links (ICLs) (1,2). Intrastrand adducts are formed when the platinum binds between purines on the same strand of DNA, and ICLs are formed when the platinum binds between guanines on opposing strands of DNA (1). Nucleotide excision repair (NER), homologous recombination, translesion synthesis (TLS) and Fanconi anemia (FA) are DNA repair pathways capable of removing cisplatin ICLs (1). Carboplatin and oxaliplatin are derivatives of cisplatin and form the same types of DNA adducts. Carboplatin is structurally the same as cisplatin once bound to DNA, as they only differ by their chemical leaving group. Oxaliplatin has a 1,2-diaminocyclohexane instead of the two amines in cisplatin and carboplatin, resulting in a different distortion to the DNA helix than those induced by cisplatin or carboplatin (3). Cisplatin or carboplatin ICLs force helical distortions, resulting in the cytosines that were bound to the guanines to become extrahelical (4). This extrahelical structure is specific to cisplatin and carboplatin, as oxaliplatin ICLs do not induce extrahelical cytosines. We have previously shown preferential oxidative deamination of the extrahelical cytosines, resulting in a uracil adjacent to the ICL, which can subsequently activate base excision repair (BER) (5). In this pathway, uracil is removed by uracil DNA glycosylase (UNG), apurinic/apyrimidinic endonuclease 1 (APE1) cleaves the phosphodiester bond near the 5′ end of the apurinic/apyrimidinic (AP) site and DNA polymerase β (Polβ) synthesizes new DNA (5–7). We have previously shown that Polβ has low fidelity and tends to misincorporate bases adjacent to the ICL (5). These mismatches can lead to the recruitment of mismatch repair (MMR) proteins (5). In this mechanistic model, the binding of BER and MMR proteins adjacent to the ICL prevents NER, homologous recombination and other productive cisplatin ICL DNA repair pathways from accessing and removing the ICL. This futile cycle of non-productive repair by BER and MMR results in ICLs persisting on the DNA, which ultimately increases the sensitivity of cells to cisplatin.

There are several cytosine deaminases that could deaminate the extrahelical cytosines formed by cisplatin ICLs, including activation-induced cytosine deaminase (AID) and the apolipoprotein B mRNA editing enzyme, catalytic polypeptide-like 3 (APOBEC3, A3) family of enzymes. There are seven members in the A3 family, denoted by APOBEC3A (A3A), APOBEC3B (A3B), APOBEC3C (A3C), APOBEC3D (A3D), APOBEC3F (A3F), APOBEC3G (A3G) and APOBEC3H (A3H). A3s occur in tandem on chromosome 22 and are thought to arise from gene duplication of primordial A3, with sequence homology ranging from 30% to 100% depending on the gene region of the A3s (8–11). A3s are antiviral cytidine deaminases that deaminate retroviruses and other viruses as part of the host defense mechanism to mutate and degrade viral genomes (12–18). Although the deamination of retroviral genomic cytidines is thought to be their primary function, A3s can also deaminate host-cell genomic cytosines resulting in the formation of uracils in DNA. The A3 family has recently been implicated as a driver of cancer development and the mutagenesis pattern of these enzymes has been found in several cancer types (8,9). Recent studies showed that A3s increase DNA mutation rate, effectively accelerating tumor development and cancer evolution (10,19). A3s have a specific mutational signature that correlates with the increase in C:G to T:A and C:G to G:C mutations in TC sequence context, which have been identified in many types of cancers, including breast cancer cohorts (10,20–23).

In the United States, breast cancer accounts for an estimated 30% of all new cancer diagnoses in women (24). Although strides in detection and treatment have improved survival, breast cancer remains the second leading cause of estimated cancer-related deaths in 2019 for women (24). Identifying the molecular factors underlying chemotherapy response may improve prognosis for breast cancer patients by selecting more effective therapy. Women with triple-negative breast cancer (TNBC) are in particular need of new approaches to treatment. TNBC is an aggressive breast cancer subtype with poor prognosis. As a breast cancer subtype characterized by the lack of three receptors, TNBCs show significant heterogeneity in treatment response and survival (25,26). There have been several recent clinical trials investigating cisplatin and carboplatin in the treatment of TNBC (27,28). In TNBC patients, treatment with carboplatin in combination with paclitaxel, doxorubicin (Dox) and bevacizumab had a 59% complete pathologic response compared to 38% in patients who did not receive carboplatin (28). In another trial, TNBC patients were treated with cisplatin following surgery. Pathologic complete response was achieved in 22% of patients, 64% had a clinical complete or partial response and 14% had disease progression (27).

Given our previous work in identifying BER and MMR mediation of cisplatin response, we hypothesized that A3 enzymes deaminate the extrahelical cytosine formed by cisplatin ICLs, thus activating BER and MMR and blocking ICL DNA repair (5–7,29). In this study, we investigated the role of A3s in mediating cisplatin, carboplatin and oxaliplatin efficacy. Our results are consistent with individual A3 family members activating BER and MMR to mediate cisplatin and carboplatin response through blocking ICL DNA repair. Based on these results and considering increased A3B expression has been linked to breast cancer, the treatment of TNBCs that express high levels of A3s with cisplatin may improve response and patient survival.

## MATERIALS AND METHODS

### Chemicals

Cisplatin, carboplatin and oxaliplatin were purchased from Sigma–Aldrich and diluted in 1× phosphate-buffered saline (PBS) for a stock concentration of 1 mM. Stock solutions were vortexed until drug was completely dissolved, followed by filtration through 0.2-μm filters. Cisplatin and carboplatin were prepared fresh for each experiment. Oxaliplatin was stored at −80°C and used within 6 months. IFNα-2b was purchased from Sigma–Aldrich (catalog no. SRP4595) and dissolved in sterile diethyl pyrocarbonate (DEPC) water to a concentration of 100 μg/ml and stored at −20°C. Phytohemagglutinin (PHA) was purchased from Sigma–Aldrich (catalog no. L8754) and dissolved in 1× sterile PBS and stored at −20°C. ERCC1 antibody was purchased from Abcam (ab76236) and GAPDH antibody was purchased from Santa Cruz (sc32233). For western blots, 50 μg/ml of total protein was utilized and a 1:1000 ERCC1 antibody dilution was used and 1:10 000 dilution for GAPDH was used.

### Cell lines

MDA-MB-231 cells were grown in RPMI containing 10% fetal bovine serum (FBS) and 1% penicillin/streptomycin. SK-BR-3 and HEK293T cells were grown in DMEM containing 10% FBS and 1% penicillin/streptomycin.

### shRNA transfection

Mission shRNA plasmid bacterial stocks targeting human MSH6, Polβ, UNG, A3B, A3C and AID were purchased from Sigma–Aldrich. Maxi prep kit (Qiagen) was used to purify the plasmid DNA. HEK293T cells were used to package the lentiviral particles using packaging plasmids PMD2G, PMDLG/RRE and PRSV/RRE. Lipofectamine 2000 (Invitrogen) was used to transfect the plasmid DNA. Twenty-four hours post-transfection, the medium was changed and viral particles were harvested 72 h post-transfection, followed by centrifugation and filtration through 0.2-μm filters. Viral stocks were aliquoted and stored at −80°C. Polybrene (Sigma–Aldrich) was used with the viral stocks to transfect cells to knock down the protein of interest. Cells were used 72 h post-transduction for the associated experiments and to check for transcript expression. Stable shPolβ cells were selected using puromycin.

### siRNA transfection

ON-TARGET plus siRNAs for human A3A, A3C, A3D and A3G and the non-targeting control siRNA were purchased from Dharmacon (siA3C target sequence: GGCAAUGUAUCCAGGCACA; siA3D target sequence: CCAAACGUCAGUCGAAUCA). siA3B and siA3F were purchased from Integrated DNA Technologies (siA3B target sequence: UCAGAUACCUGAUGGAUCCA). For siA3F, 5′-GAACCAAUCUCUGCUAAUUUUUCTA-3′ and 5′-UAGAAAAAUUAGCAGAGAUUGGUUCUG-3′ were utilized. Transfection was carried out as per Dharmacon’s protocol. Cells were plated in a six-well plate without penicillin/streptomycin. Transfection was completed with 60–70% cell density, with two transfections performed 24 h apart. DharmaFECT 4 transfection reagent was used for MDA-MB-231. The cells were used 48 h after the initial transfection for the associated experiments and to assess transcript expression.

### Quantitative real-time PCR

Cells were harvested and pelleted. RNA was extracted using TRIzol (Invitrogen) using standard procedures. RNA concentration was determined using SpectraMax M5. Two micrograms of total RNA was reverse transcribed using High-Capacity cDNA Reverse Transcription Kit (Applied Biosystems). Transcript levels were then quantified using PowerUP SYBR Green Master Mix (Applied Biosystems), with GAPDH as an endogenous control. The transcript changes were determined from 2^−ΔΔCT^ values, done in triplicate and repeated per associated experiment. A3 primer sequences were obtained from (9).

### Colony survival assay

Four hundred to one thousand cells were seeded in 60-mm dishes, with cell number varying by cell line. Cells were treated with increasing concentrations of cisplatin, carboplatin or oxaliplatin for 2 h in serum-free media. Following treatment, complete medium was added and the cells were allowed to grow for 7–14 days at 37°C with 5% CO_2_. Colonies were fixed and stained with 0.2% crystal violet in 20% ethanol. Colonies with ≥50 cells were counted and colony survival was expressed as the ratio of the average number of colonies in drug-treated cells compared to untreated cells multiplied by 100. The experiment was done in biological and technical triplicates for each drug concentration. IC_50_ values were calculated using CompuSyn version 1. For experiments with IFNα-2b, the concentration utilized was 0.5 μg/ml and the concentration of PHA was 2 μg/ml.

### Modified alkaline comet assay

Modified alkaline comet assay was used to analyze the repair of ICLs as previously described (5–7,29). Following drug treatment and time course for ICL DNA repair, cells are collected and incubated with control buffer or fresh hydrogen peroxide. The hydrogen peroxide incubation results in DNA single-strand and double-strand breaks that can be separated using alkaline buffers and agarose gel electrophoresis. The use of hydrogen peroxide and induction of DNA strand breaks after cell harvest serve only to fragment the DNA. The presence of covalently linked ICLs under alkaline conditions retards the DNA migration of the fragmented DNA, whereas repair of the ICLs results in faster migration that can be detected in the comet tail moment. Cell suspensions containing ∼10 000 cells were embedded on a microscope slide in agarose, lysed and incubated in cold alkaline buffer for 20 min to allow for the DNA to unwind. Electrophoresis was performed for 25 min at 300 mA and 20–25 V. Slides were neutralized, and then stained with SYBR3 Gold (Invitrogen). The comets were scored using a Nikon fluorescence microscope. At least 50 cells were analyzed per slide using ImageJ (1.52k) and OpenComet v1.3 plugin (30). Data are expressed as the percent mean olive tail moment, which corresponds to the percent remaining ICLs.

### *In vitro* ICL activity assay

The oligonucleotide substrate (5′-CTCTTCCCCCTCTCCTTCTTGCCCTCTTCCTTCCCCTTCCCT-Cy3-3′) was treated with aquated cisplatin to create a single site-specific ICL as previously described (6). This substrate has a single guanine for the formation of a mono-adduct. Briefly, following platination of the oligonucleotide, it was annealed to the complementary oligonucleotide (3′-GAGAAGGGGGAGAGGAAGAACGGGAGAAGGAAGGGGAAGGGA-5′), followed by overnight dialysis at 37°C in 100 mM sodium perchlorate and 10 mM Tris–HCl, pH 7.5, and room temperature for 8 h to form the ICL. The ICL was then ethanol precipitated, purified by DNA sequencing gel, excised from the gel, eluted and ethanol precipitated to obtain the cisplatin ICL oligonucleotide substrate. For the incision reactions, A3 incision buffer (20 mM Tris, pH 8.0, 10 mM MgCl_2_ and 1 mM 2-mercaptoethanol) and DNA substrate were incubated with 500 ng of purified A3 protein (A3B-CTD or A3C) for 2 h at 37°C, which was followed by the addition of UDG and APE1 for an additional 45 min. The reaction products were either loaded directly or ethanol precipitated and then separated on 12% sequencing gels.

### Purification of A3C

A3C was cloned into pAB-6xHis baculovirus vector (AB Vector). Sf9 insect cells were transfected using ProFectin (AB Vector), ProGreen (AB Vector) and pAB-6xHis-A3C. Baculovirus was propagated following the manufacturer’s protocol. Sf9 insect cells were infected with P4 baculovirus, and E64 proteinase inhibitor (Sigma–Aldrich) was added 24 h post-infection. Sf9 cells were lysed with 20 mM HEPES, pH 7.5, buffer with added pepstatin, leupeptin and PMSF, 150 mM NaCl, 10 mM NaF, 10 mM sodium phosphate, 10 mM EDTA, 1 mM imidazole, 10 mM DTT, 10% glycerol and 1% Triton X-100. Cells were kept on ice for 20 min, vortexing two to three times during incubation. Cells were then sonicated at 60% amp three times for 15 s with 30 s in between. The lysed cells were then centrifuged and the supernatant added to an equilibrated nickel column. The column was washed with 50 mM HEPES and 200 mM NaCl and 6xHis-A3C eluted in fractions using 50 mM HEPES, 150 mM NaCl, 200 mM imidazole and 1% glycerol.

### Overexpression of A3s

We utilized the Tet-On 3G tetracycline-inducible expression system (TaKaRa) to assess the effect of overexpression of individual A3 proteins on drug response. A3C, A3D and A3G were individually cloned into pTRE3G. Lentivirus was generated of the pTRE3G plasmids and Tet-On 3G transactivator containing plasmid. SK-BR-3 cells were utilized for overexpressing individual A3 proteins due to the low expression of A3s in these cells. SK-BR-3 cells were infected with both pTRE3G (with A3C, A3D or A3G) and Tet-On 3G. G418 (Tet-On 3G) and puromycin (pTRE3G) were used to select constitutively expressing cells unless transient expression of the A3s was utilized for the experiments. Cells were grown as described above using Tet-free FBS (Atlanta Biologicals) and Dox (0.5 μg/ml) was added to activate expression of A3C, A3D or A3G.

### TCGA breast cancer dataset

Breast cancer data from The Cancer Genome Atlas (TCGA) were obtained from cBioPortal (http://www.cbioportal.org). Male breast cancer cases (*n* = 12) and metastatic site samples (*n* = 7) were excluded from analysis. Estrogen receptor (ER) and progesterone receptor (PR) status was determined using immunohistochemistry (IHC). HER2 status was determined using IHC or fluorescence *in situ* hybridization if IHC results were not available (31). Subtype was determined by ER, PR and HER2 status: TNBC cases were negative for each receptor, HER2-enriched cases were positive for HER2 and negative for ER/PR, and Luminal cases were positive for ER or PR.

### Statistical analysis

All data have a minimum of three technical and biological replicates and outcomes (such as IC_50_ value, percent mean olive tail moment at each time point and RNA expression level by RT-PCR) were summarized with means and standard deviations. For statistical hypothesis testing, the distribution of each outcome was checked for normality assumption and, if needed, a data transformation was applied. Unpaired two-sided *t*-tests were used to determine significance between two groups and three or more groups were compared using one-way ANOVA followed by Tukey’s post-hoc analysis. RNA Seq V2 RSEM values were used for gene expression analysis and transformed by *z*-score (i.e. scaled to a mean of 0 and standard deviation of 1). Distributions of overall survival (OS) for each gene were summarized using Kaplan–Meier curves and estimates between low and high expressions separated by the median expression level. Survival curves were statistically compared between groups using a log-rank test. Univariable and multivariable Cox proportional hazard regression analyses were performed with prechosen covariates (age, TNM and subtype) previously associated with OS. The proportional hazards assumption was checked and no violation was found. All data analyses were carried out using R version 3.5.0, RStudio: Integrated Development for R (version 1.1.447, RStudio Inc., Boston, MA) and SigmaPlot version 10.0.

## RESULTS

### Expression of A3s in cell line models

The expression of each A3 in SK-BR-3 and MDA-MB-231, the two main cell lines used in this manuscript, is shown in Supplementary Figure S1A. SK-BR-3 cells have lower expression of each A3, except A3A, when compared with MDA-MB-231 cells (Supplementary Figure S1A). MDA-MB-231 cells have nearly undetectable A3A levels; thus, A3A expression was not included in subsequent graphs assessing A3A expression in these cells. SK-BR-3 cells express low levels of A3A but have undetectable A3B; thus, A3B expression was not included in subsequent graphs assessing A3B levels in these cells.

### Resistance to cisplatin and carboplatin with knockdown of A3s

**Figure 1.**
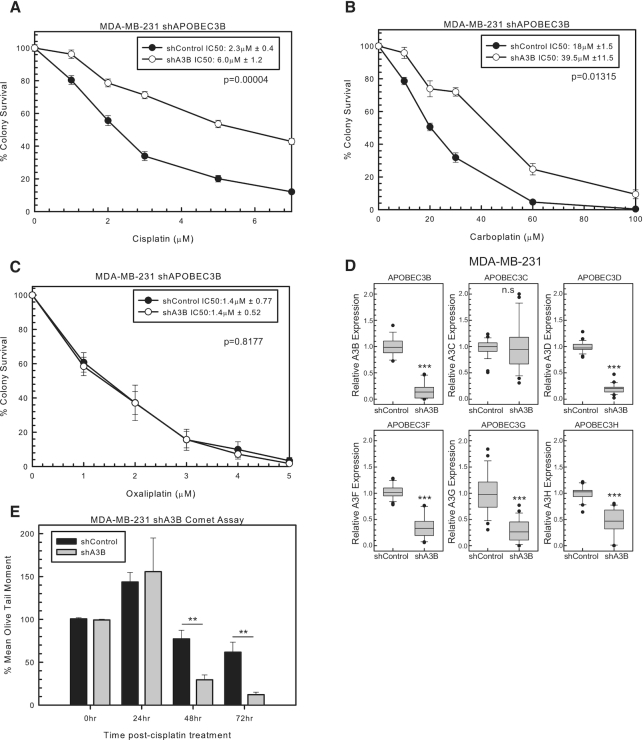
A3 mediation of cisplatin and carboplatin response. MDA-MB-231 colony survival assay with shControl or shA3B treated for 2 h with (**A**) cisplatin, (**B**) carboplatin and (**C**) oxaliplatin. *P*-values determined using unpaired two-sided *t*-test. (**D**) MDA-MB-231 A3 expression determined by RT-PCR for shControl or shA3B. Unpaired two-sided *t*-test was used to determine *P*-values. (**E**) Modified alkaline comet assay in MDA-MB-231 with shControl or shA3B treated for 2 h with 10 μM cisplatin. Error bars are standard deviation. n.s. represents not significant, *P < 0.05, **P < 0.01 and ***P < 0.001.

A3B is linked to breast cancer development and has high relative expression in MDA-MB-231 cells. We, therefore, used shRNA to A3B to determine whether A3B expression influences cisplatin response. shA3B resulted in resistance to cisplatin and carboplatin compared to shControl in colony survival assays (Figure 1A and B, respectively). In contrast, the oxaliplatin ICL structure does not form extrahelical cytosines to the same extent (32–34). Consistent with this lack of availability of extrahelical cytosine in oxaliplatin ICLs, shA3B did not alter oxaliplatin response compared to shControl (Figure 1C). RT-PCR was used to confirm A3B knockdown with shA3B (Figure 1D). However, due to the sequence homology between A3s, the expression of each A3 was assessed. Along with decreased A3B expression, shA3B decreased the expression of A3D, A3F, A3G and A3H. Therefore, resistance to cisplatin and carboplatin with shA3B may be attributed to any of these A3s (A3B, A3D, A3F, A3G and A3H) or due to a potential combination of A3 knockdown (Figure 1D).

### Increased ICL DNA repair with knockdown of A3s

Due to cisplatin and carboplatin resistance with shA3B, we next wanted to determine whether this effect was due to changes in ICL DNA repair. ICL removal between shControl and shA3B was determined by a modified alkaline comet assay. Twenty-four hours post-cisplatin treatment, there is an increase in ICLs in both shControl and shA3B (Figure 1E). This increase is consistent with the conversion of cisplatin mono-adducts to ICLs, as ICL formation has been shown to peak around 18 h post-treatment (Figure 1E). At 48 and 72 h post-cisplatin treatment, shA3B cells had less ICLs than shControl, suggesting that cells are able to remove ICLs faster (Figure 1E). These data suggest that resistance to cisplatin and carboplatin occurs as a result of increased ICL DNA repair in shA3B compared to shControl.

### Resistance to cisplatin and carboplatin with knockdown of A3s using shA3C

Considering shA3B results in knockdown of other A3 family members and is not specific to A3B, we assessed another shRNA, shA3C. In MDA-MB-231 cells, shA3C decreased expression of A3C, A3D, A3F, A3G and A3H (Supplementary Figure S1C). With the reduced expression of multiple A3s, shA3C cells were resistant to cisplatin consistent with what was observed with shA3B (Supplementary Figure S1B). We also knocked down AID in MDA-MB-231 cells using shAID and the level of expression following knockdown with shAID compared to shControl is shown in Supplementary Figure S1E. However, there was no difference between shControl and shAID with cisplatin treatment in colony survival assays (Supplementary Figure S1D). These results suggest that individual or combinations of A3 family members, but not AID, can mediate cisplatin and carboplatin response similar to what we have previously shown with loss of BER or MMR (5–7).

### A3 expression predicts breast cancer survival

In order to assess whether there was any clinical relevance to APOBEC3s altering breast cancer patient outcome, we utilized TCGA data (Supplementary Figure S2). A3s have been suggested to be linked to the development of breast cancer, but whether A3 expression can correlate with patient outcome has not been addressed. Stratifying by breast cancer subtype, in TNBC cases with Kaplan–Meier survival curves, high median expression of A3C, A3D or A3F correlated with better OS (*P* = 0.061, *P* = 0.043 and *P* = 0.0019, respectively; Supplementary Figure S2A). A3A, A3B, A3G and A3H were not statistically significant (Supplementary Figure S2B). Cox proportional hazard models were used to further investigate the association between A3 expression and OS. A3D and A3F were associated with better OS in the triple-negative subtype in the univariable model [hazard ratio (HR) 0.44, 95% CI 0.20–0.98, *P* = 0.045 and HR 0.31, 95% CI 0.14–0.68, *P* = 0.0034, respectively; Supplementary Table S1). In the multivariable model, A3F remained statistically significantly associated with better OS (HR 0.15, 95% CI 0.055–0.41, *P* = 0.00018; Supplementary Table S1). These data show that TNBC patients have better OS with higher expression of A3C, A3D and A3F. The correlation of high A3 expression and better OS could be linked to effects on chemotherapy response, including with cisplatin and carboplatin as well as cytoxan that can cross-link DNA at GC sequences similar to the platinum-based drugs.

### Resistance to cisplatin with knockdown of A3D

**Figure 2.**
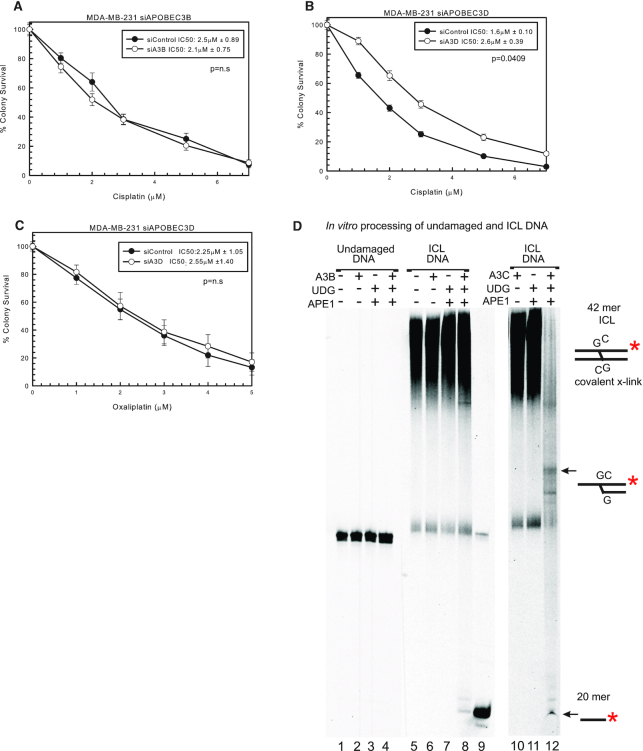
Specific A3 knockdown using siRNA to evaluate cisplatin response. (**A**) MDA-MB-231 colony survival assay with siControl or siA3B with 2-h cisplatin treatment. MDA-MB-231 colony survival assay with siControl or siA3D treated for 2 h with (**B**) cisplatin or (**C**) oxaliplatin. *P*-values determined using unpaired two-sided *t*-test. (**D**) *In vitro* deamination and incision assay. Undamaged DNA, lanes 1–4; ICL DNA, lanes 5–8 and 10–12. A3B-CTD, UDG, APE1 and A3C are signified with + if in the reaction. A 20mer marker was loaded in lane 9. Substrate depictions and possible reaction products are included next to the gel.

To further assess which A3s can mediate response to cisplatin, we used siRNAs specific to A3 family members. Using specific siRNA to A3B, there was no difference between siControl and siA3B in MDA-MB-231 cells with cisplatin treatment (Figure 2A and Supplementary Figure S3A), suggesting that the knockdown of A3B with shA3B is not responsible for the change in cisplatin response seen in Figure 1A. A3D had decreased expression with shA3B, the largest decrease after A3B (Figure 1D). Considering A3B appears not to influence cisplatin response or OS in patients, we used a specific siRNA to A3D. siA3D resulted in resistance to cisplatin compared to siControl (Figure 2B). Interestingly, there is a slight increase in expression of A3G and A3H after siA3D transfection but the MDA-MB-231 cells already have relatively high levels of these A3s expressed (Supplementary Figure S3B). There is no off-target downregulation of the other A3 family members, so this demonstrates that A3D plays a role in cisplatin response. In addition, siA3D has no effect on oxaliplatin response as expected (Figure 2C). We also tested a specific siRNA to A3A, which resulted in no change in cisplatin, carboplatin or oxaliplatin response in SK-BR-3 cells that express A3A (Supplementary Figure S3C). The knockdown level of A3A using siA3A averaged over 80% when compared to control siRNA (expression data not shown). We designed and tested other siRNAs targeting other family members (e.g. A3C, A3F and A3G), and consistent with what we observed with siA3A and siA3B, there was minimal effect on cisplatin sensitivity (Supplementary Figure S3D–F). Knockdown levels of A3C and A3G averaged >85%, while knockdown levels of A3F with two separate siRNAs averaged ∼40% (expression data not shown). These data suggest that either there was not sufficient level of knockdown of each family member individually (e.g. A3F) or potentially multiple A3 family members are required to drive the maximum response to cisplatin and carboplatin. The latter would be consistent with what we observe using shA3B and shA3C where multiple family members are downregulated to drive a drug response. In addition, even with siA3D, we only observed ∼2-fold resistance (Figure 2B). In order to achieve maximum effect in response to cisplatin and carboplatin and to help elucidate the mechanism of how A3 family members can mediate drug response, we utilized the shA3B that targets multiple A3 family members in subsequent knockdown and rescue experiments.

### *In vitro* deamination activity of purified A3B-CTD and A3C

To further assess the role of A3s in mediating cisplatin response and further test the hypothesis that certain A3 family members could deaminate the extrahelical cytosines at a cisplatin ICL, purified A3 enzymes were reacted with either undamaged double-stranded DNA or cisplatin ICL DNA substrate. We purified and tested the catalytic C-terminal domain (CTD) of A3B and full-length A3C with cisplatin ICL DNA. Following treatment with A3s, the DNA was treated further with UDG and APE1 to create a strand break at the uracils created by A3s. This A3B-CTD protein did not have any activity on undamaged double-stranded DNA (lanes 1–4, Figure 2D) (35,36). ICL DNA alone is shown in lane 5 as a low-mobility smear, as the cross-linked DNA runs slower through the gel compared to undamaged DNA. There is no strand cleavage with A3B-CTD or UDG and APE1 incubated individually (lanes 6 and 7, Figure 2D). A3B-CTD incubation with the ICL followed by incubation with UDG and APE1 shows a very small amount of product corresponding to a 20mer compared to the control 20mer size marker in lane 9. This is a representative gel and repeated experiments yielded similar results (Supplementary Figure S4). For the A3B-CTD, increased reaction times did not result in increased reaction products (data not shown).

When purified A3C was used instead of A3B-CTD, incubation of ICL DNA with this enzyme followed by incubation with UDG and APE1 shows near-complete conversion of cytosines at the ICL to uracil and only a residual smear at the top of the gel and a strong 20mer band in lane 12 (Figure 2D). There are also additional bands seen, suggesting that A3C may be deaminating the bottom DNA strand cytosine as well as the top strand. There is loss of fluorescent signal relative to the control input DNA levels that are likely the result of limitations of the fluorophore detection limit of certain reaction products. A3C had minimal to no activity on undamaged duplex DNA (Supplementary Figure S4, lanes 1–4). In addition, following enzyme incubation and subsequent DNA ethanol precipitation to eliminate potential salt effects on product electrophoresis, A3C can deaminate additional cytosines that are 3′ to the extrahelical cytosine adjacent to the cross-linked guanine in the ICL DNA substrate (Supplementary Figure S4, lane 8, products below 20-mer). These data suggest that A3B-CTD has minimal activity with the ICL, and that A3B-CTD is not as efficient as A3C. These data are consistent with the colony survival data that demonstrated that specific A3B knockdown had no effect on cisplatin response and the lack of correlation between A3B expression and OS in breast cancer patients. These data are also consistent with the hypothesis that A3 family members can deaminate the extrahelical cytosine adjacent to the cisplatin ICL, which helps drive the specific response to cisplatin and carboplatin.

### Cisplatin resistance with combination knockdowns of BER, MMR and A3s

**Figure 3.**
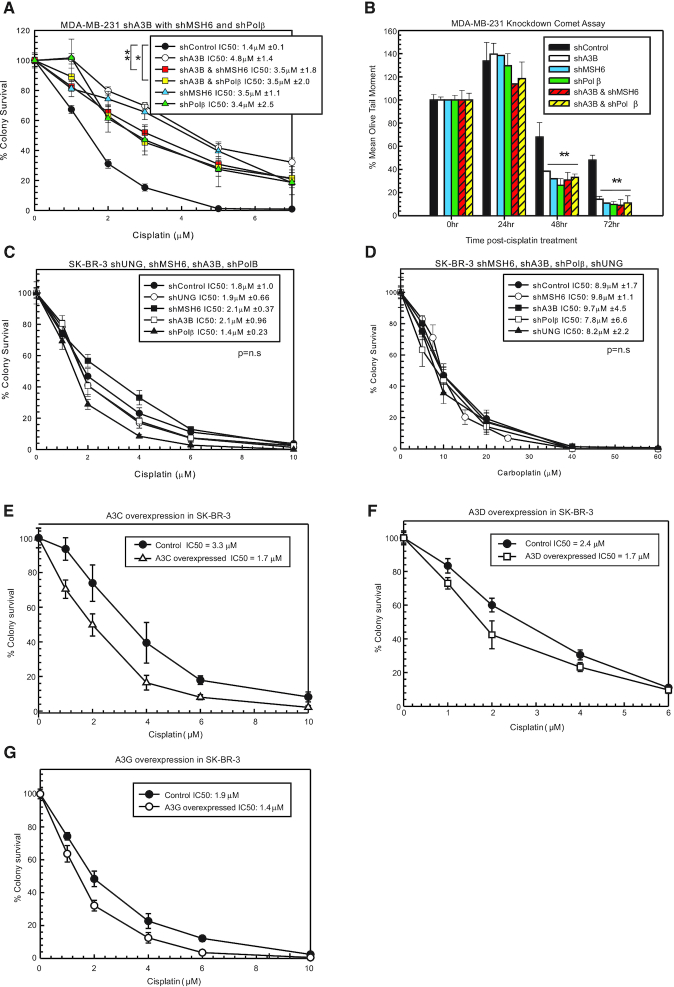
Epistatic relationship of A3 enzymes, BER and MMR to mediate cisplatin response. (**A**) MDA-MB-231 colony survival assay with 2-h cisplatin treatment with shControl, shA3B, shA3B and shMSH6, shA3B and shPolβ, shMSH6 and shPolβ. (**B**) Modified alkaline comet assay with the cells from (A) treated for 2 h with 10 μM cisplatin. Colony survival assay with SK-BR-3 shControl, shUNG, shMSH6, shA3B and shPolβ with 2-h (**C**) cisplatin or (**D**) carboplatin treatment. Colony survival assays in SK-BR-3 cells with cisplatin treatment following overexpression of (**E**) A3C, (**F**) A3D or (**G**) A3G in SK-BR-3 cells compared to control constructs. Colony survival *P*-values determined using unpaired two-sided *t*-test. n.s. represents not significant, *P < 0.05, **P < 0.01 and ***P < 0.001.

To confirm that A3s mediate cisplatin response and act within the same pathway as BER and MMR, we used combination knockdowns in MDA-MB-231 cells. shA3B knockdown with shMSH6 or shPolβ had the same resistance to cisplatin (e.g. within the standard deviation margins) as individual knockdowns of shA3B, shMSH6 or shPolβ (Figure 3A). These data suggest that A3s activate BER and subsequently MMR, as the combination loss did not increase resistance to cisplatin. In addition, combination knockdown of shA3B with shMSH6 or shPolβ had the same resistance to carboplatin (Supplementary Figure S5A). shA3B with shMSH6 or shPolβ, along with shA3B alone, shMSH6 and shPolβ, did not alter oxaliplatin response as expected (Supplementary Figure S5B).

A modified alkaline comet assay was used to determine whether resistance to cisplatin and carboplatin was due to changes in ICL DNA repair. ICLs increased in all samples at 24 h post-cisplatin treatment (Figure 3B), consistent with what was observed in Figure 1E. At 48 and 72 h time points, shA3B, shMSH6 and shPolβ, as well as the combination of shA3B with shMSH6 or shPolβ, had less percent remaining ICLs compared to shControl (Figure 3B). These data suggest that without A3s and active BER (Polβ) and MMR (MSH6), cells have faster cisplatin ICL repair, and that A3s are acting in the same mechanistic pathway and are epistatic with BER and MMR to mediate cisplatin and carboplatin response.

### Alterations in cisplatin response by BER and MMR require A3 expression

Another breast cancer cell line, SK-BR-3, has significantly lower expression of A3s compared to MDA-MB-231 cells (Supplementary Figure S1A). In this cell line, knockdown of UNG, MSH6, Polβ or A3s using shRNA did not increase resistance to cisplatin, carboplatin or oxaliplatin compared to control (Figure 3C and D, and Supplementary Figure S5D, respectively). Based on the hypothesis, we speculated the lack of effect on cisplatin response is due to the low expression of A3s in SK-BR-3, as without the initial deamination of the extrahelical cytosine, BER and MMR will not be activated and will not block functional removal of the ICLs. The A3 knockdown levels of each shRNA are shown in Supplementary Figure S5E. SK-BR-3 cells have low expression of each A3 except for A3A. Downregulation of A3A in SK-BR-3 cells had no effect on cisplatin response (Supplementary Figure S3C), suggesting that A3A is not mediating drug response. When A3C, A3D and A3G were individually overexpressed transiently or by Dox induction in SK-BR-3 cells, there was modest increased sensitivity to cisplatin (Figure 3E–G, respectively). There was similar modest increased sensitivity to carboplatin with individual A3C, A3D and A3G overexpression while not effecting oxaliplatin sensitivity (data not shown). An additional cell line that has low A3 expression, HEK293T, also does not have altered cisplatin, carboplatin or oxaliplatin response with knockdown of UNG, MSH6 or A3B (Supplementary Figure S6A–C). Knockdown levels following each shRNA are shown in Supplementary Figure S6D. These data suggest that A3s are required to activate BER and MMR to mediate cisplatin and carboplatin response.

### Induction of A3 expression sensitizes cells to cisplatin and carboplatin

**Figure 4.**
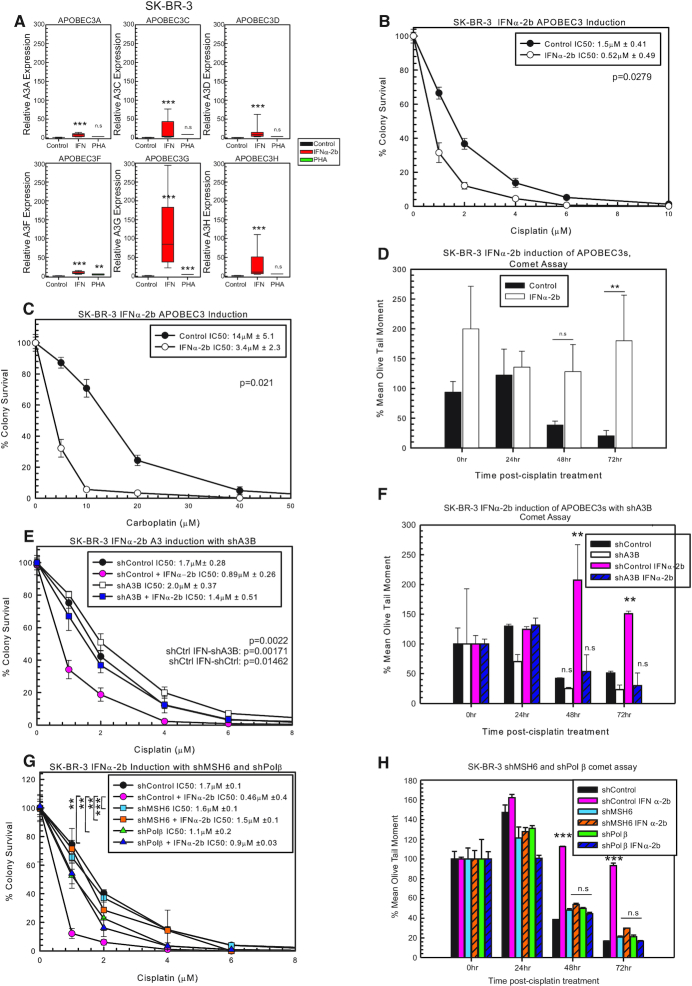
Induction of A3s with IFNα-2b and sensitization to cisplatin treatment. (**A**) A3 expression in SK-BR-3 determined by RT-PCR. *P*-values determined using unpaired two-sided *t*-test. SK-BR-3 colony survival assay with control or IFNα-2b treatment with 2-h (**B**) cisplatin or (**C**) carboplatin treatment. (**D**) Modified alkaline comet assay with SK-BR-3 control or IFNα-2b-treated cells with 2-h 5 μM cisplatin treatment. (**E**) SK-BR-3 colony survival assay with shControl or shA3B with or without IFNα-2b with 2-h cisplatin treatment. *P*-values determined using one-way ANOVA with Tukey’s post-hoc analysis. (**F**) Modified alkaline comet assay with the cells from (E) after 2-h 5 μM cisplatin treatment. (**G**) SK-BR-3 colony survival assay with shControl, shMSH6 and shPolβ with or without IFNα-2b with 2-h cisplatin treatment. *P*-values determined using one-way ANOVA with Tukey’s post-hoc analysis. (**H**) Modified alkaline comet assay with cells from (G) after 2-h 5 μM cisplatin treatment. n.s. represents not significant, *P < 0.05, **P < 0.01 and ***P < 0.001.

Due to modest cisplatin and carboplatin drug response effects following individual A3 knockdown or individual overexpression of A3 family members, we hypothesized that multiple A3 family members are required to drive maximum drug response. In order to further investigate this possibility, we sought to test compounds that can globally induce A3 family expression in cell line models that have low levels of A3 family members. There are several compounds that increase A3 expression, with varying expression in different cell lines (9,10,37–43). In SK-BR-3 cells, IFNα-2b treatment increases the expression of each A3, whereas PHA did not significantly increase A3 expression in this cell line (Figure 4A). Pre-treatment with IFNα-2b sensitizes SK-BR-3 to cisplatin and carboplatin treatment (Figure 4B and C, respectively). IFNα-2b treatment did not alter oxaliplatin response in SK-BR-3 cells (Supplementary Figure S7A). These data suggest that with the induced expression of A3s, BER and MMR are activated and sensitize cells to cisplatin. We utilized the modified alkaline comet assay to determine whether the sensitization seen with IFNα-2b is due to blocking ICL DNA repair. At 48 and 72 h post-cisplatin treatment, SK-BR-3 IFNα-2b-treated cells had more percent remaining ICLs compared to control (Figure 4D). At 72 h, cells should remove a majority of the ICLs; however, in the IFNα-2b-treated cells, there is no removal of the ICLs, suggesting that the increase in A3 expression is activating BER and MMR, which prevent the removal of the ICLs via blocking productive ICL DNA repair pathways (Figure 4D).

IFNα-2b can induce the expression of many genes within cells (37–42), and to determine whether the changes in cisplatin response and ICL removal are specific to A3s, we combined shA3B with IFNα-2b treatment to rescue the effect and reduce A3 expression to control levels (Supplementary Figure S7B). As expected, shControl cells treated with IFNα-2b were sensitive to cisplatin, while there was no difference between shControl, shA3B and shA3B with IFNα-2b (Figure 4E). This suggests that the sensitivity to cisplatin seen with IFNα-2b treatment is specifically due to increased A3 expression. In support of this, the modified alkaline comet assay with shControl with IFNα-2b did not result in removal of ICLs compared to shControl, shA3B and shA3B with IFNα-2b, which all demonstrated faster ICL DNA repair (Figure 4F). There were also no additional effects observed between shA3B alone or in combination with IFNα-2b, suggesting that IFNα-2b alters ICL removal by the induction of A3s. In addition, IFNα-2b treatment in SK-BR-3 or MDA-MB-231 cells does not alter expression of a critical cisplatin DNA repair factor (ERCC1), which further supports the role of APOBEC3 induction as the mechanism in IFNα-2b inducing cisplatin sensitivity (Supplementary Figure S7C).

To determine whether these data are linked to BER and MMR activation, we combined IFNα-2b treatment with shMSH6 or shPolβ. As shown previously, shControl with IFNα-2b was sensitive to cisplatin (Figure 4G). The shMSH6 and shPolβ in combination with IFNα-2b resulted in resistance to cisplatin (Figure 4G). There was no difference in cisplatin response between shControl, shMSH6, shMSH6 with IFNα-2b, shPolβ and shPolβ with IFNα-2b (Figure 4G). In addition to the colony survival data, there was no difference between shControl, shMSH6 with or without IFNα-2b and shPolβ with or without IFNα-2b in the modified alkaline comet assay (Figure 4H). These results support that IFNα-2b induction of A3s activates BER (Polβ) and MMR (MSH6) to mediate cisplatin response and the resistance can be rescued with shA3B, shMSH6 or shPolβ (Figure 4G and H). Along with the combination knockdown results observed in MDA-MB-231 cells, these A3 induction and subsequent knockdown results strongly support an epistatic relationship between A3s, BER and MMR pathways to mediate drug response to cisplatin and carboplatin.

PHA treatment in HEK293T cells induces A3 expression, while IFNα-2b does not significantly increase expression (Supplementary Figure S7D). In HEK293T cells, A3 induction with PHA pre-treatment sensitizes cells to cisplatin and carboplatin compared to control (Supplementary Figure S7E and F, respectively). PHA pre-treatment did not alter oxaliplatin response (Supplementary Figure S7G), suggesting that in HEK293T cells, induction of A3s allows for deamination of the cisplatin and carboplatin extrahelical cytosines, therefore specifically sensitizing cells to cisplatin and carboplatin. Modified alkaline comet assay showed that at 48 and 72 h post-cisplatin treatment, PHA-treated cells had more percent remaining ICLs compared to control (Supplementary Figure S7H). These data suggest that when A3s are expressed, ICL removal is prevented by activating BER and MMR similar to what was observed in SK-BR-3 cells. To further determine whether PHA or IFNα-2b cause off-target changes within the cells that alters cisplatin response, we treated MDA-MB-231 cells with PHA or IFNα-2b. MDA-MB-231 cells already have high A3 expression and there is minimal increase to A3 expression with treatment (Supplementary Figure S7J). There was no difference between control, PHA- or IFNα-2b-treated cells with cisplatin in colony survival assays in the MDA-MB-231 cells as they already express sufficient A3 levels to affect cisplatin and carboplatin response. These data demonstrate that A3 expression induction is responsible for the enhanced sensitivity to cisplatin and carboplatin and not off-target effects (Supplementary Figure S7I).

## DISCUSSION

We have previously shown that loss of BER and MMR drives resistance to cisplatin and carboplatin, and the activation of BER in this response is dependent on UNG activity (5,7). In addition, this resistance is due to enhanced ICL DNA repair (5–7). Considering the requirement for UNG in this mechanistic model, we speculated that a cytosine deaminase would be required to initiate oxidative deamination of the extrahelical cytosines induced by cisplatin and carboplatin ICLs. A3 enzymes can deaminate cytosines and have been linked to cancer; thus, we speculated that this family of cytosine deaminases may be critical for uracil formation at the ICLs and subsequent activation of BER to maintain cisplatin sensitivity. In this study, our results demonstrate that specific A3 family members can influence the response to cisplatin and carboplatin as well as correlate with OS in breast cancer patients. Loss of A3s through shRNA results in resistance to cisplatin and carboplatin, and resulted in faster ICL DNA repair consistent with what we have previously shown with loss of BER or MMR (5–7). Expression of A3C, A3D or A3F correlated with better OS in TNBC, and focusing on these members, we show that specific knockdown of A3D results in resistance to cisplatin and carboplatin. Using an *in vitro* deamination assay with a cisplatin ICL DNA substrate, we find that A3C can deaminate the extrahelical cytosines and activate BER. In addition, overexpression of individual A3 family members (e.g. A3C, A3D and A3G) in SK-BR-3 cells that have low A3 levels can drive modest cisplatin sensitivity. We highlight that these data support an epistatic relationship between A3s, BER and MMR to mediate cisplatin response, as knockdown of A3s with BER (Polβ) or MMR (MSH6) combined did not increase resistance beyond the individual knockdowns. The enhanced rate of ICL DNA repair is also consistent with these combination knockdowns, suggesting that A3s act within the same mechanistic pathway as BER and MMR to mediate the response to both cisplatin and carboplatin. Inducing global A3 expression in cell line models that have intrinsically low A3 levels using either IFNα-2b or PHA resulted in significant sensitivity to cisplatin and carboplatin, as well as inhibited ICL DNA repair. Although interferons have multiple targets, we find that this response is specific to our proposed model, as knocking down A3s in combination with IFNα-2b treatment negated the sensitivity seen with IFNα-2b alone. In addition, A3 induction by IFNα-2b and subsequent knockdown results with A3s, BER and MMR proteins strongly support the epistatic connection between these pathways in mediating cisplatin and carboplatin response.

**Figure 5.**
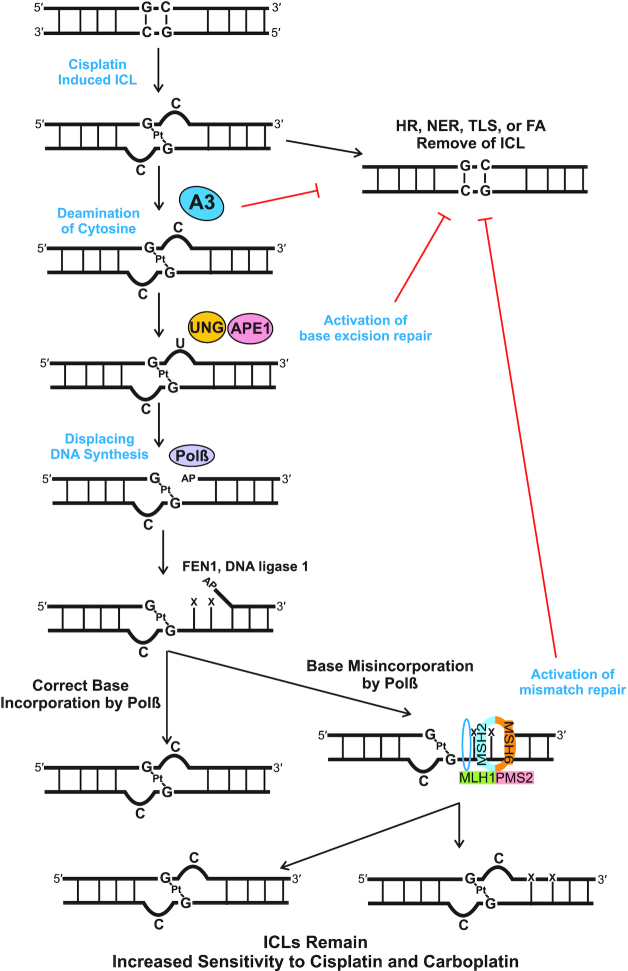
Model of A3 activation of BER and MMR. Our data are consistent with A3 family members deaminating the extrahelical cytosine at cisplatin and carboplatin ICLs. The subsequent uracil induced by the A3s activates the BER pathway, specifically via UNG recruitment followed by APE1 incision at the abasic site. Polβ synthesizes DNA downstream of the ICL and preferentially misincorporates nucleotides, which leads to recruitment of the MMR proteins. The subsequent futile cycle of processing the flanking DNA at the ICLs results in blocking productive ICL DNA repair and leads to enhanced sensitivity to cisplatin and carboplatin.

Together, these data highlight the role A3 enzymes play in mediating cisplatin and carboplatin response. These data highlight that multiple A3 family members can mediate the response to cisplatin and carboplatin and that multiple family members are required for maximum response. We previously identified that oxidative deamination of the extrahelical cytosine activates BER (UNG, APE1 and Polβ) and subsequent MMR (MSH2, MSH6, MLH1 and PMS2) (5–7,29). We propose a mechanistic model to explain the interaction of A3s, BER and MMR pathways in mediating this drug response (Figure 5). Cisplatin binds to two guanines on opposing strands of DNA, causing the cytosines that were bonded to the guanines to become extrahelical. A3s then deaminate the extrahelical cytosines, resulting in uracil formation and activation of BER. UNG removes the uracil formed by A3s and APE1 cleaves the DNA backbone, generating an AP site for Polβ. Polβ then synthesizes new DNA, FEN1 removes the flap generated and DNA ligase 1 seals the DNA gap. Polβ has low fidelity, and we have previously shown that adjacent to the ICL, Polβ tends to misincorporate bases (5). MMR proteins MutSα (MSH2 and MSH6) and MutLα (MLH1 and PMS2) then initiate either correct or incorrect repair of the mismatched bases (5,6). Each of these steps has enzymes acting adjacent to the ICL, which physically prevents enzymes from NER, homologous recombination, TLS or FA from accessing the ICL. This non-productive cycle of repair results in the ICLs remaining in the DNA, resulting in apoptosis and increasing cisplatin and carboplatin sensitivity.

Determining the specific A3 enzymes capable of the deamination of the extrahelical cytosines adjacent to cisplatin ICLs is still under active investigation and likely involves the combination of a couple of A3 family members including A3C, A3D, A3F and A3G. Our results indicate that the cytosine deaminase AID has no effect on cisplatin response, suggesting a specific requirement for A3 family members in mediating drug response. A3s have been shown to have activity on single-stranded DNA; however, we have speculated that the helical distortion caused by the cisplatin ICL may offer a substrate for A3s to deaminate the extrahelical cytosine in double-stranded DNA. Recent publications have shown that some A3s distort the helix similar to that of the ICL during deamination (44–46); however, this has been shown in processive A3s and has not been investigated in non-processive A3s (47).

Type I interferons induce the expression of interferon-stimulated genes, including STAT1/2, SOCS and A3s. IFNα induces A3s through the type I IFN receptor and activation of PKC and STAT1, and inhibition of PKC prevents the induction of A3B in breast cells (37–42,48,49). IFNα treatment is used in treating chronic hepatitis B and recent cell culture studies have shown that induction of A3 and BER by IFNα treatment is essential for decreasing hepatitis B virus (39,50–52). Although A3 deamination activity can activate BER and MMR, there is no evidence for IFNα-2b or A3 expression inducing expression of ICL DNA repair pathway proteins (39,50,51). IFNα-2b is clinically approved to treat chronic hepatitis B, hairy cell leukemia, malignant melanoma, follicular lymphoma, and chronic and acute hepatitis C. IFNα has also been utilized in the treatment of other cancer types. In clinical trials in pancreatic ductal adenocarcinoma using IFNα in combination with radiation, 5-fluorouracil (5-FU) and cisplatin, patients had increased 2- and 5-year survival (53–55). Seventy-three percent of epidermoid carcinoma patients and 33% of adenocarcinoma advanced esophageal carcinoma patients responded when treated with IFNα, 5-FU and cisplatin (56). IFNα combined with methotrexate, 5-FU and cisplatin in locally advanced head and neck cancer had 6.25% partial response and 46.7% partial response (57). Interferon treatment has also been tested in breast cancer with mixed results [reviewed in (58)]. Most of these studies have been done with small sample size, often only in advanced or metastatic disease, and not done in prospective randomized trials.

Future directions of this work will focus on further testing the mechanistic model and elucidating how A3s (specifically A3C, A3D, A3G and A3F) mediate cisplatin response. Current research suggests differential localization for these enzymes. A3C has been shown to be cytoplasmic and nuclear, while A3D and A3F have been shown to be cytoplasmic (40,59–63). A3D is a double-domain deaminase and expression of either the N-terminal or C-terminal domain can localize to the nucleus, which raises the possibility that protein alteration could mediate cellular localization (64). A3G has been shown to localize to the nucleus following ionizing radiation; thus, in response to chemotherapeutic agents, A3 cellular localization may also be altered (65). It will be interesting to determine whether A3 nuclear localization occurs in response to cisplatin treatment or following dysregulation and whether A3C, A3D, A3G and A3F are capable of deaminating the extrahelical cytosine formed by cisplatin ICLs in genomic DNA.

Based on our data, IFNα may improve patient response to cisplatin treatment by inducing A3 expression, activating BER and MMR to block productive ICL repair, therefore preventing the removal of the ICLs and enhancing cisplatin response (Figure 5). In addition, further studies looking at patient samples and A3 expression levels and how they correlate with chemotherapy response may help elucidate whether specific A3 family members may prove useful as biomarkers for therapeutic response, including cisplatin and carboplatin. Recent published literature is consistent with our results and highlights a role for A3 expression correlating with patient response to platinum agents in ovarian as well as head and neck cancers (66,67). Our novel studies have provided a mechanistic understanding of how APOBEC3 family members can influence cisplatin and carboplatin drug response and potentially be useful as biomarkers to predict platinum drug response.

## Supplementary Material

zcaa033_Supplemental_File
