# Supplementary material for: APOBEC3 enzymes mediate efficacy of cisplatin and are epistatic with base excision repair and mismatch repair in platinum response
Source: NAR Cancer. 2020 Nov 6;2(4):zcaa033. doi: 10.1093/narcan/zcaa033 (PMC7646253; doi:10.1093/narcan/zcaa033)

SUPPLEMENTARY DATA

Supplementary Table 1. Univariable and multivariable cox proportional analysis of A3 expression in relation to overall survival in breast cancer

	**Univariable[a]**		**Mulitvariable[b]***		**Univariable: Triple-Negative cases[c]**		**Multivariable: Triple Negative cases[d]****	
**Gene (Median)**	**HR (95% CI)**	**p-value†**	**HR (95% CI)**	**p-value†**	**HR (95% CI)**	**p-value†**	**HR (95% CI)**	**p-value†**
A3A	0.87 (0.64, 1.1)	0.25	0.89 (0.60, 1.3)	0.58	0.76 (0.33, 1.8)	0.52	0.83 (0.32, 2.2)	0.71
A3B	0.86 (0.65, 1.1)	0.28	0.98 (0.67, 1.5)	0.92	1.2 (0.49, 2.8)	0.73	1.3 (0.50, 3.5)	0.57
A3C	1.04 (0.78, 1.4)	0.78	0.80 (0.54, 1.2)	0.27	0.45 (0.19, 1.1)	0.068	0.43 (0.17, 1.1)	0.075
A3D	0.69 (0.52, 0.91)	0.0093	0.55 (0.37, 0.81)	0.0024	0.44 (0.20, 0.98)	0.045	0.56 (0.23, 1.4)	0.21
A3F	0.81 (0.61, 1.1)	0.13	0.60 (0.40, 0.88)	0.01005	0.31 (0.14, 0.68)	0.0034	0.15 (0.06, 0.41)	0.00018
A3G	0.73 (0.55, 0.97)	0.031	0.64 (0.43, 0.93)	0.02023	0.74 (0.32, 1.7)	0.48	0.56 (0.23, 1.4)	0.21
A3H	0.62 (0.47, 0.83)	0.0011	0.62 (0.42, 0.90)	0.013	1.0 (0.45, 2.3)	0.97	0.95 (0.39, 2.3)	0.91

Numbers included in analysis: [a]1184 [b]874 [c]142 [d]121

*Multivariable: Diagnosis Age, Tumor Size, Lymph Node Infiltration, Metastasis, ER, PR, HER2
**Multivariable: Diagnosis Age, Tumor Size, Lymph Node Infiltration, Metastatsis
†Walt Test Statistic

SUPPLEMENTARY DATA

Supplementary Figures Legends

Supplementary Figure 1. MDA-MB-231 cells with shControl and shA3C. a. RNA Sequencing (RPKM) expression of A3s in MDA-MB-231 and SK-BR-3. RNA-Seq data from the Cancer Cell Line Encyclopedia, downloaded from cbioportal. **b**. colony survival assay with two-hour cisplatin treatment **c**. RT-PCR of shControl and shA3C of each A3 **d**. shControl and shAID colony survival assay with two-hour cisplatin treatment. **e**. RT-PCR of shControl and shAID. Colony survival and RT-PCR p-values determined using unpaired two-sided t-test. n.s=not significant, *p<0.05, **p<0.01, ***p<0.001.

Supplementary Figure 2. Overall survival by median expression for TNBC patients in TCGA. Kaplan-Meier survival curves by high or low median for **a.** A3C, A3D and A3F expression and **b.** A3A, A3B, A3G, or A3H expression; p-values determined by log-rank test.

Supplementary Figure 3. RT-PCR of MDA-MB-231 cells to assess A3 expression and A3A knockdown in SK-BR-3 cells. siControl with **a**. siA3B and **b**. siA3D. **c** Colony survival in SK-BR-3 cells following downregulation of A3A. **d** Colony survival in MDA-MB-231 cells following downregulation of A3C. **e** Colony survival in MDA-MB-231 cells following downregulation of A3F. **f** Colony survival analysis in MDA-MB-231 cells following downregulation of A3G. p-values determined using unpaired two-sided t-test. n.s=not significant, *p<0.05, **p<0.01, ***p<0.001.

Supplementary Figure 4. *In vitro* deamination and incision assay. DNA was incubated with enzymes and DNA products subsequently ethanol precipitated prior to sequencing gel electrophoresis. Undamaged DNA lanes 1-4, and ICL DNA lanes 5-8 incubated with purified A3C, UDG and APE1 where signified with + if in the reaction. 20mer marker was loaded in lane 9. Substrate depictions and possible reaction products are included next to the gel.

Supplementary Figure 5. Epistatic role of A3s, BER and MMR in carboplatin and oxaliplatin response. Colony survival assays using shControl, shA3B, shMSH6, shPolβ, shA3B & shMSH6, shA3B & shPolβ treated for two hours with **a**. carboplatin or **b**. oxaliplatin and **c**. RT-PCR of A3s, MSH6, and Polβ. SK-BR-3 cell colony survival assay with shControl, shMSH6, shA3B, shPolβ, and shUNG **d**. treated for 2 hours with oxaliplatin and **e.** RT-PCR results of each knockdown. Colony survival and RT-PCR p-values determined using unpaired two-sided t-test. n.s=not significant, *p<0.05, **p<0.01, ***p<0.001.

Supplementary Figure 6. HEK293T colony survival and expression analysis. shControl, shUNG, shMSH6, and shA3B colony survival assay treated for two hours with **a**. cisplatin **b.** carboplatin and **c** oxaliplatin. **d**. RT-PCR of A3s, MSH6 and UNG of each knockdown. Colony survival and RT-PCR p-values determined using unpaired two-sided t-test. n.s=not significant, *p<0.05, **p<0.01, ***p<0.001.

Supplementary Figure 7. Assessment of IFNα-2b and PHA effects on cisplatin, carboplatin and oxaliplatin response. a. Colony survival assay with SK-BR-3 treated with IFNα-2b and two hour oxaliplatin treatment. **b**. RT-PCR of SK-BR-3 with shControl and shA3B with or without

IFNα-2b of each A3. **c.** Western blot of ERCC1 protein in SK-BR-3 and MDA-MB-231 cells either untreated or with IFNα-2b. GAPDH is for loading controls. **d**. RT-PCR of HEK293T cells with control, PHA or IFNα-2b treatment of each A3. HEK293T colony survival assay with PHA pre-treatment with two hour treatment with **e**. cisplatin **f**. carboplatin and **g.** oxaliplatin. **h.** modified alkaline comet assay with PHA pre-treated HEK293T cells after 10μM cisplatin treatment. **i**. MDA-MB-231 colony survival assay with control, PHA or IFNα-2b pre-treatment with two-hour cisplatin treatment. **j**. RT-PCR of MDA-MB-231 PHA and IFNα-2b treated cells of each A3. Colony survival and RT-PCR p-values determined using unpaired two-sided t-test. n.s=not significant, *$p<0.05$, **$p<0.01$, ***$p<0.001$.

Supplementary Figure 1.

a

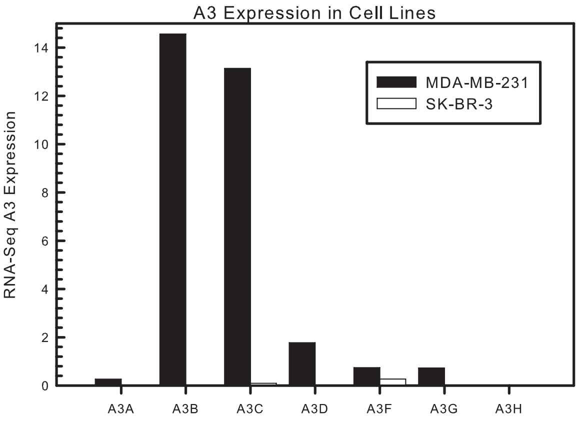

b

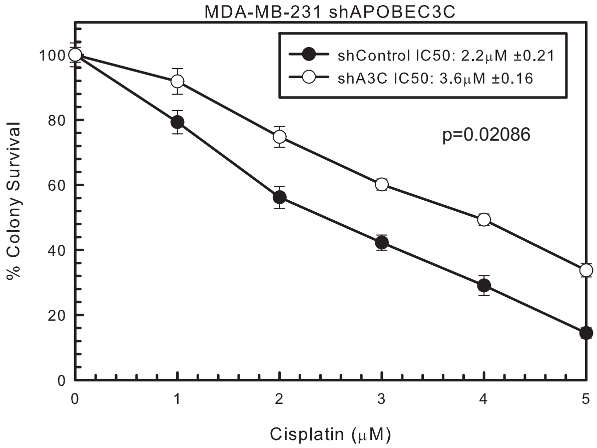

c

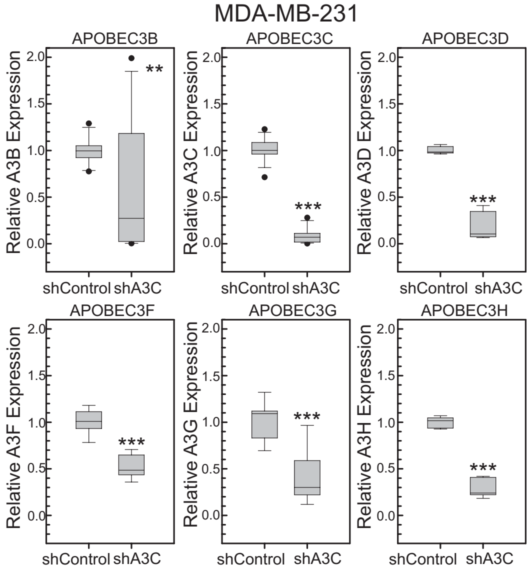

d

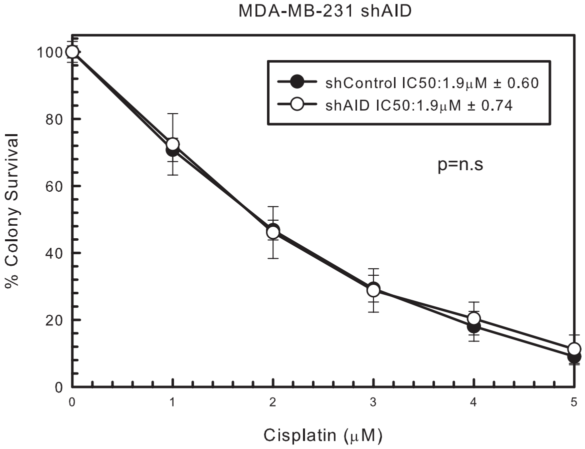

e

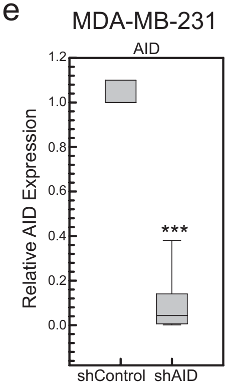

Supplementary Figure 2.

a

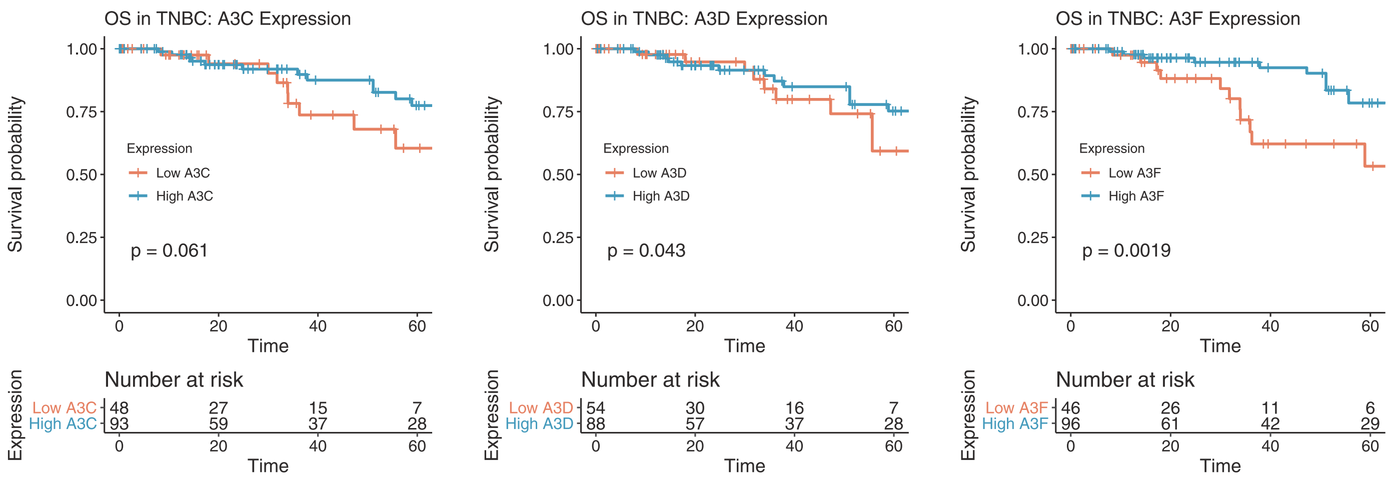

b

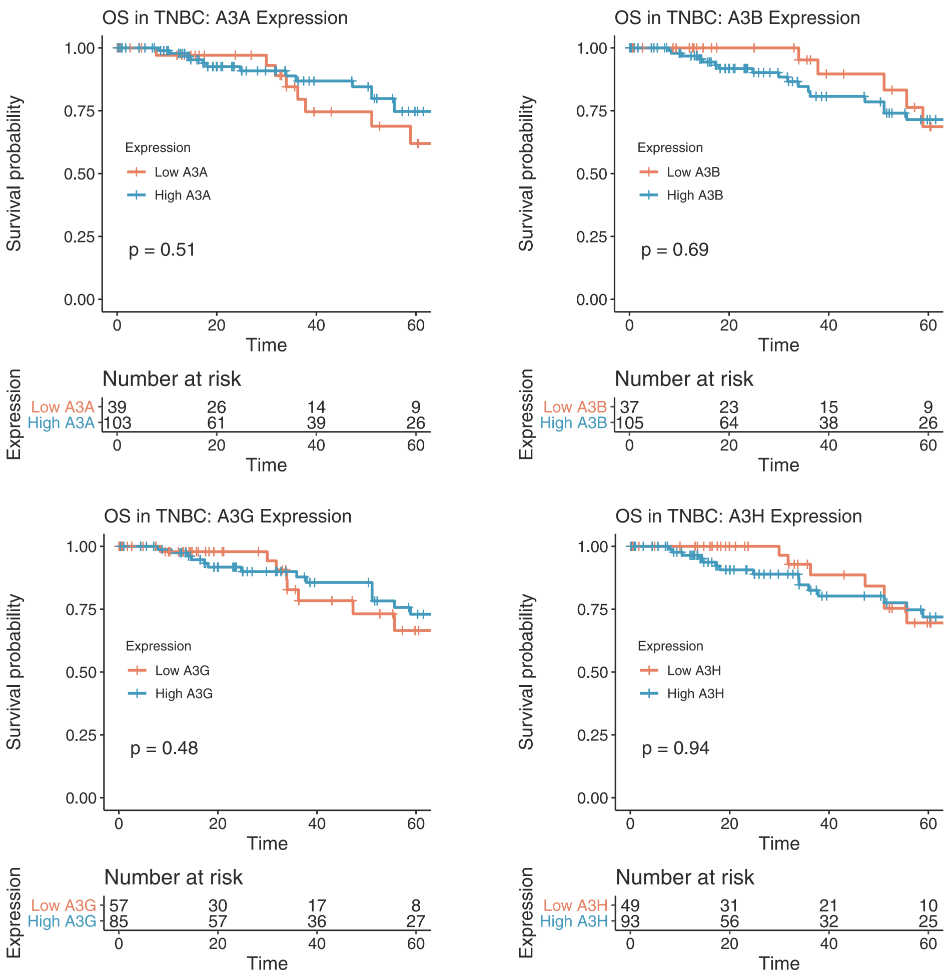

Supplementary Figure 3.

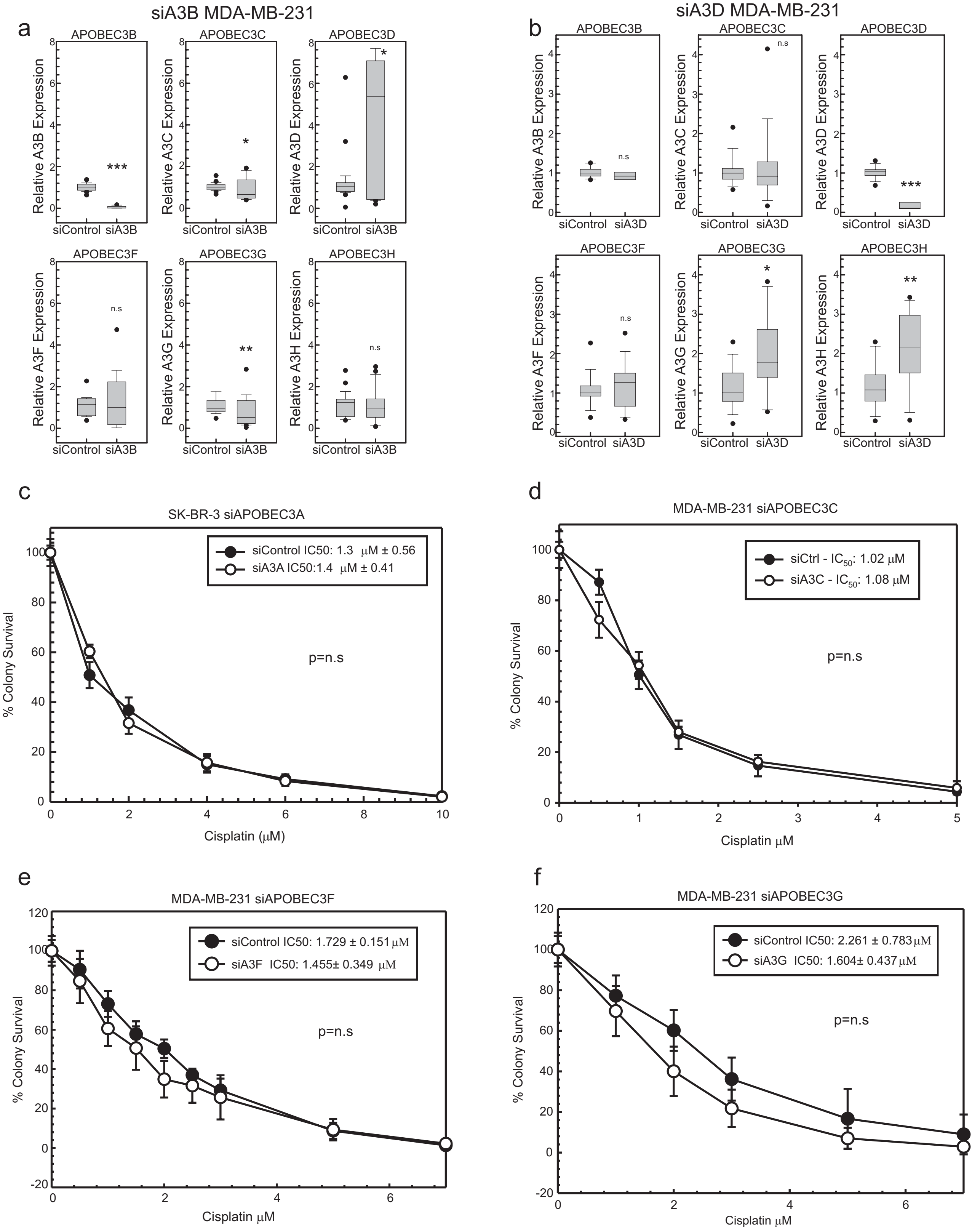

Supplementary Figure 4.

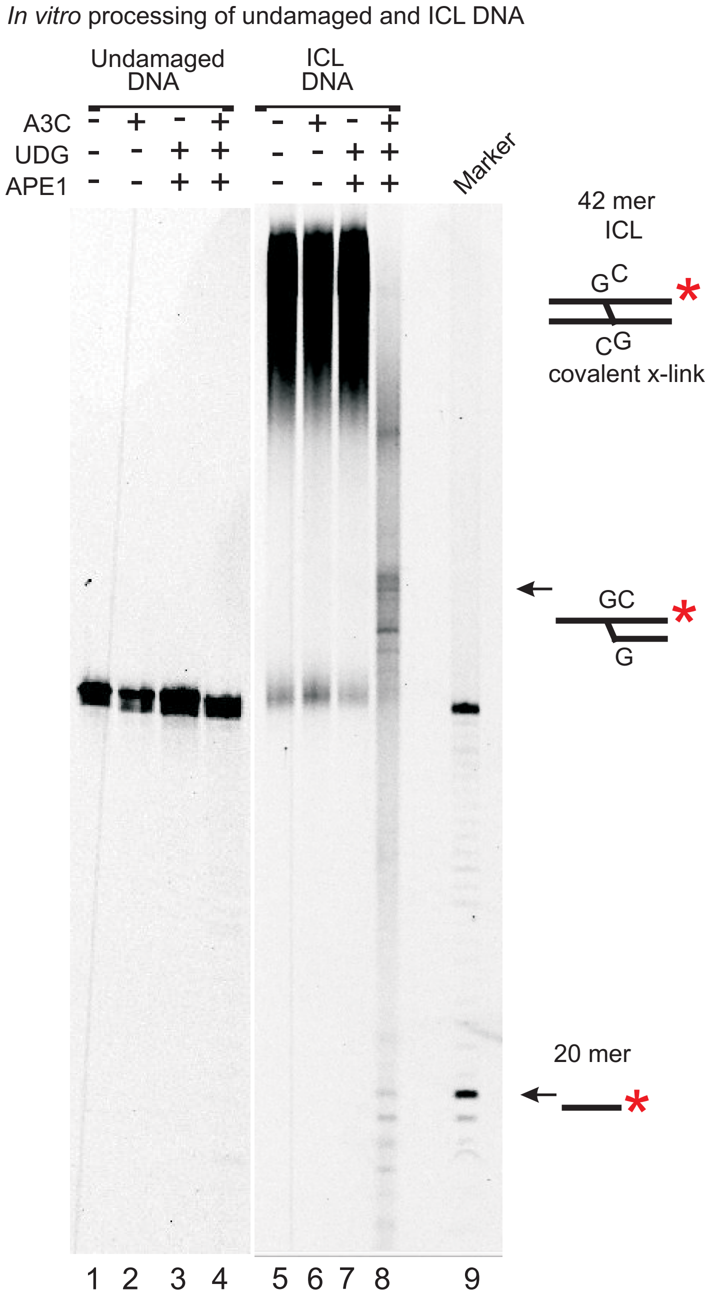

Supplementary Figure 5.

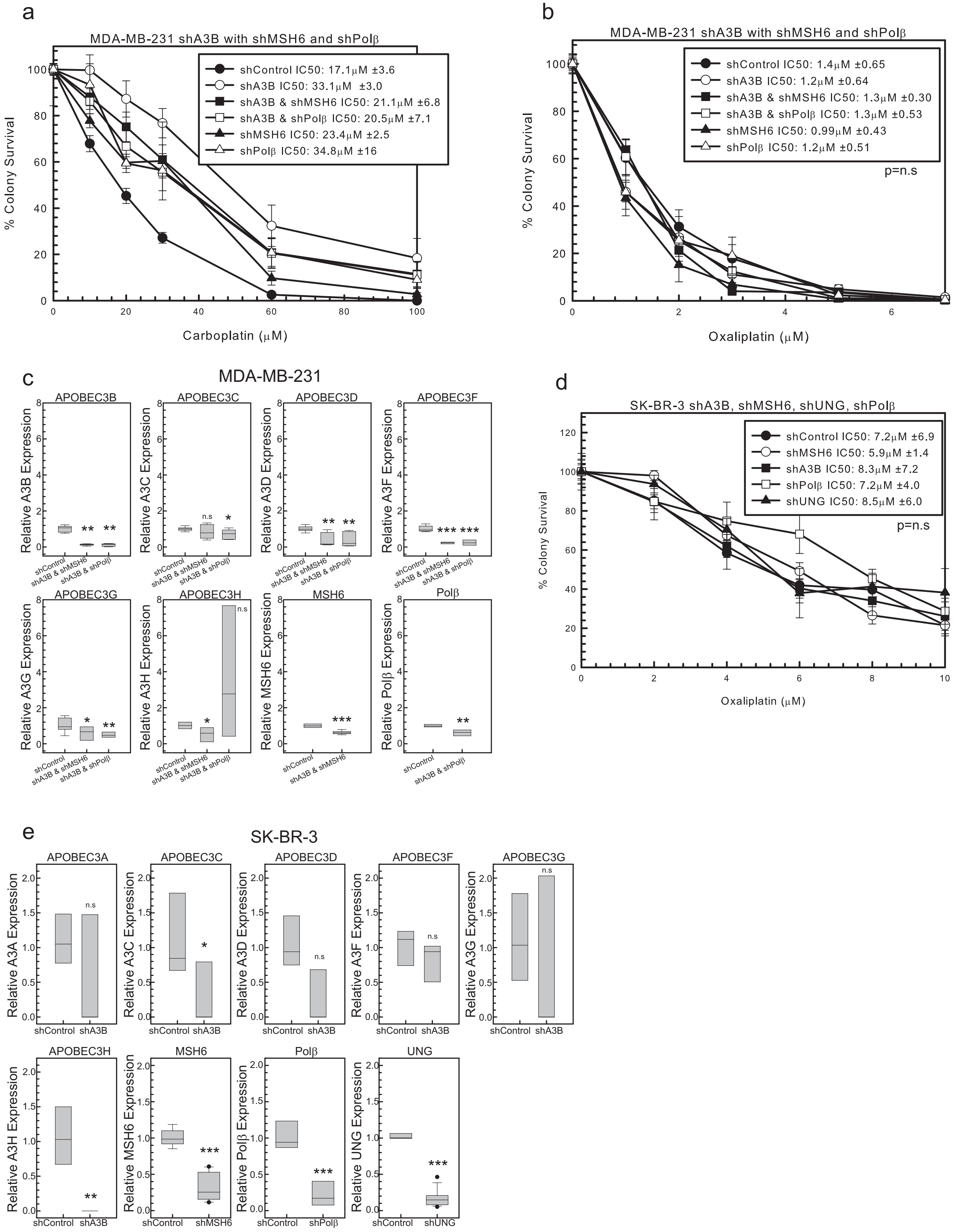

Supplementary Figure 6.

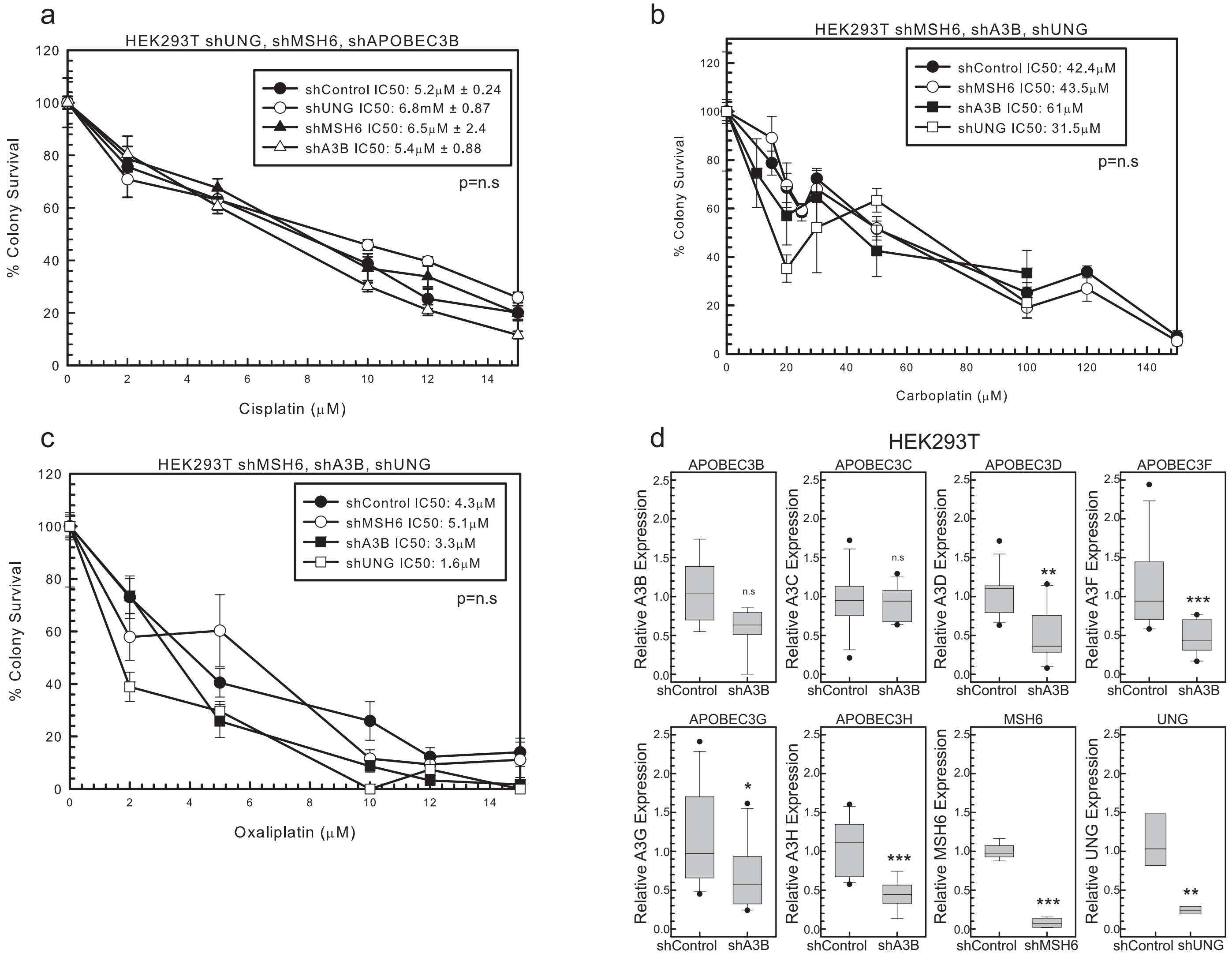

Supplementary Figure 7.

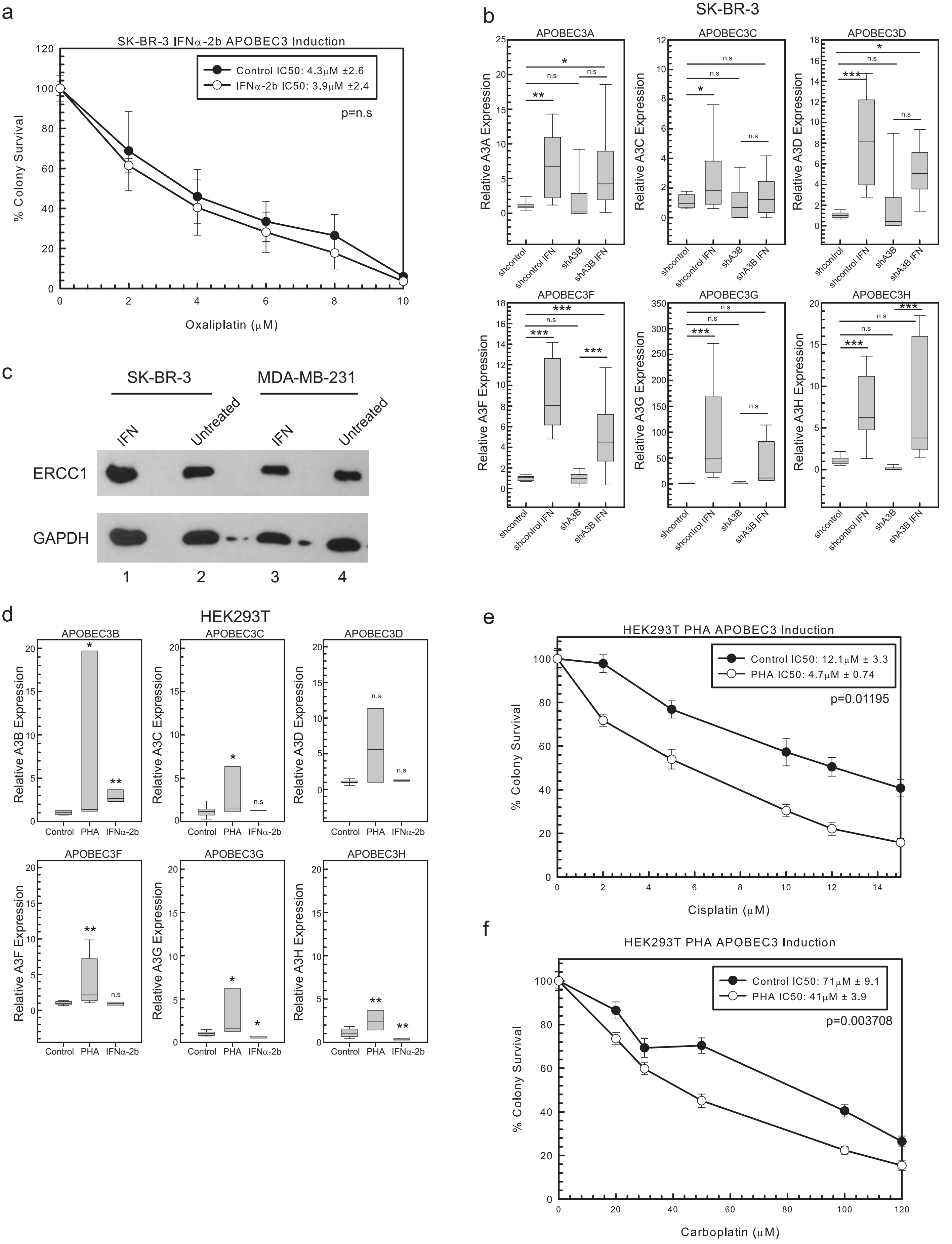

Supplementary Figure 7.

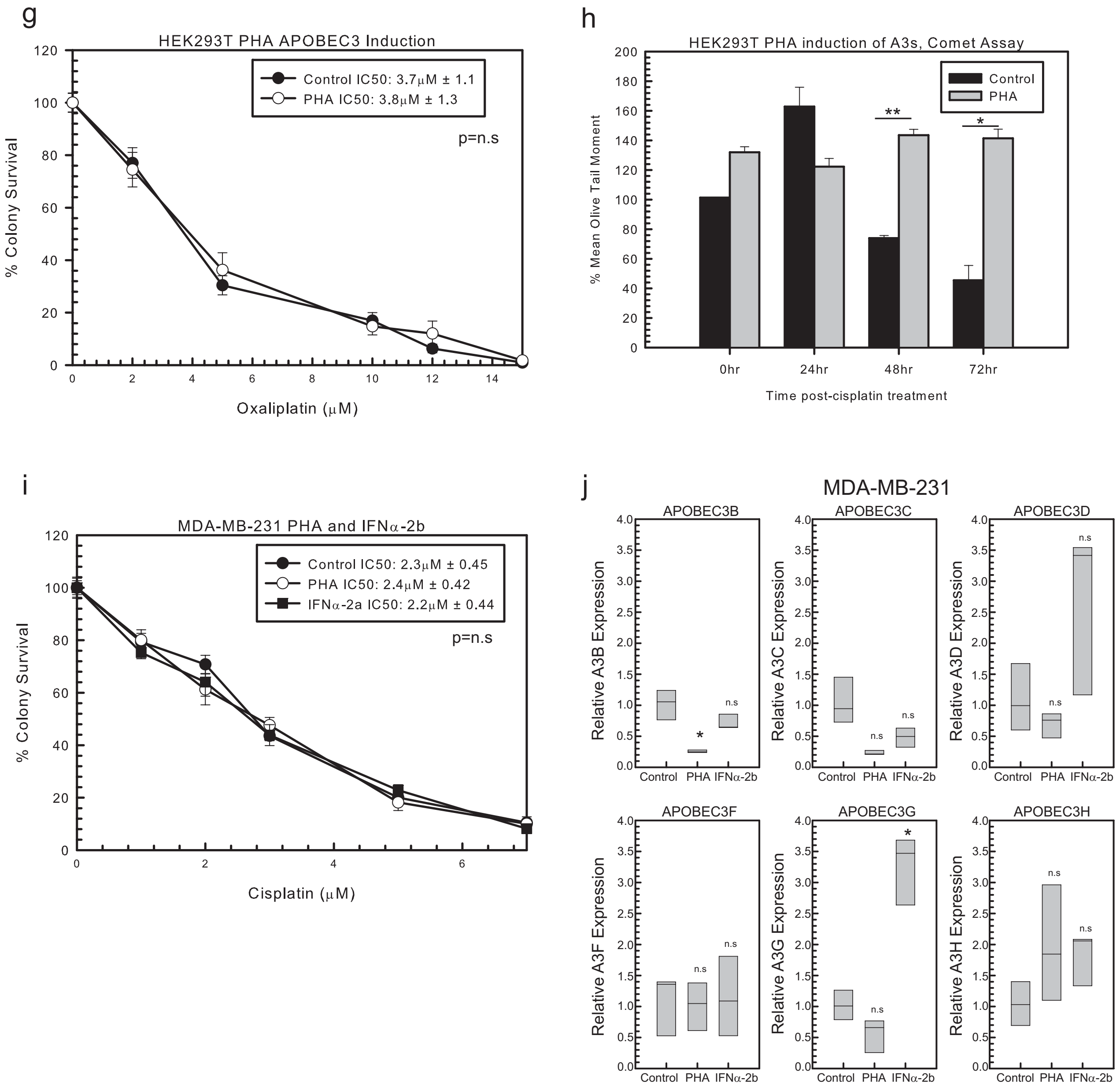

Supplement: zcaa033_Supplemental_File [file zcaa033_supplemental_file.pdf]
